# Salt Formation of the Alliance of Triazole and Oxadiazole Towards Balanced Energy and Safety

**DOI:** 10.3390/ma18153435

**Published:** 2025-07-22

**Authors:** Yang Liu, Meiqi Wang, Jiawei Men, Bibo Li, Shangbiao Feng, Shuangfei Zhu, Guangrui Liu, Ruijun Gou, Shuhai Zhang, Ming Lu, Li Yang

**Affiliations:** 1 School of Environmental and Safety Engineering, North University of China, Taiyuan 030051, China; liuyang2160@nuc.edu.cn (Y.L.); 18636155886@163.com (M.W.); menjiawei1023@163.com (J.M.); 13194237617@163.com (B.L.); fengshb@nuc.edu.cn (S.F.); zsf0201@nuc.edu.cn (S.Z.); guangruiliu@nuc.edu.cn (G.L.); 2 School of Chemistry and Chemical Engineering, Nanjing University of Science and Technology, Nanjing 210094, China; luming@njust.edu.cn; 3 Shanxi Jiangyang Chemical Co., Ltd., Taiyuan 030041, China; yl13754892849@163.com

**Keywords:** hydrogen-bonded organic frameworks, energetic materials, detonation performances, thermal stability

## Abstract

Balancing the energy and stability of energetic materials is a challenging task in their development. Salt formation is a promising strategy for seeking high-energy, low-sensitivity materials. In this study, the modification of anions facilitates the enhancement of density and oxygen balance in amino-functionalized N-heterocycle systems. The results of single-crystal X-ray diffraction and theoretical analysis suggest that DATOP possesses intense hydrogen bonding networks in its crystal structure. The ideal structure of DATOP (*ρ* = 1.954 g·cm^−3^, *D* = 8624 m·s^−1^, *P* = 34.4 GPa) gives rise to higher detonation properties compared to DATOC (*ρ* = 1.717 g·cm^−3^, *D* = 5984 m·s^−1^, *P* = 12.4 GPa). In particular, the thermal stability of DATOP (*T_d_* = 273 °C) is superior to DATOC (*T_d_* = 154 °C). DATOP also maintains comparable mechanical sensitivities to DATOC. These fascinating results reveal that the strategy of salt formation shows excellent potential for balancing energy and stability in energetic materials.

## 1. Introduction

Energetic materials are metastable compounds that can rapidly release much energy through self-redox reactions, which raise considerable concerns in the military and civilian fields [1,2,3]. Many milestone substances, such as 2,4,6-trinitrotoluene (TNT), 1,3,5-trinitro-1,3,5-triazinane (RDX), 1,3,5,7-tetranitro-1,3,5,7-tetrazocane (HMX), 2,4,6,8,10,12-hexanitro-2,4,6,8,10,12-hexaazaisowurtzitane (CL-20), have been developed and used on a large scale for their excellent comprehensive properties [4,5,6]. Nowadays, high-energy, low-sensitivity explosives have become a long-term goal due to increasing energy and safety demands. However, high energy level for energetic materials tends to poor molecular stability [7,8].

The traditional strategy for the construction of energetic materials is intramolecular integration of fuel and oxidant elements. In recent decades, the intermolecular self-assembly of fuel and oxidant components has become an effective method for exploring advanced energetic materials, such as energetic cocrystals [9], metal–organic frameworks (MOFs) [10,11], and molecular perovskite energetic materials [12,13,14]. Furthermore, salt formation emerge as a promising strategy for balancing energy and safety of energetic materials [15,16,17], and hydrogen bonds can affect the sensitivity of compounds through their influence on electrostatic potential [18,19]. For example [20], TKX-50 is a star energy salt with a high detonation performance (*D* = 9698 m·s^−1^, *P* = 42.4 GPa) and good sensitivity (IS = 20 J, FS = 120 N) demonstrating the superiority of energetic salts to adjust the properties of explosives.

High-nitrogen heterocycles have advancements of high enthalpy of formation, good stability, rendering them ideal structure units for high-energy, low-sensitivity materials. A number of N-heterocyclic energetic materials have been reported, such as 2,2′-dinitramino-5,5′-bi (1,3,4-oxadiazole) (ICM-101), 4-amino-3,7-dinitrotriazolo-[5,1-c][1,2,4]triazine-4-oxide (DPX-27), 3,3′-dinitroamino-4,4′-azoxyfurazan, and pentazolate salts [21]. The energetic alliance of 1,2,4-triazole and 1,3,4-oxadiazole was investigated as a promising skeleton for bearing energetic functional groups. Herein we subjected 2-amino-5-(5-amino-1*H*-1,2,4-trizol-3-yl)-1,3,4-oxadiazole (DATO) to a self-assembly process with inorganic guest molecules to understand the pivotal role of salt formation in modulating the properties of energetic materials.

## 2. Experimental Section

***Caution!*** All the materials are potential explosives. Necessary safety protection must be taken, such as face shield, eye-protecting glasses, gloves, and coats. Intense mechanism action should be avoided while handling these energetic compounds.

DATOC: DATO (167 mg, 1 mmol) was added in 10 mL water, and then 2 mL of 36% hydrochloric acid was dropwise added under stirring. The system was further stirred for 1 h and was placed at room temperature to crystallize. After one day, colorless block-shaped crystals were filtered. The yield is 84%. ^1^H NMR (600 MHz, DMSO-*d*_6_): δ = 8.35 ppm. ^13^C NMR (151 MHz, DMSO-*d*_6_): δ = 162.63, 154.51, 149.05, 140.57 ppm. IR (KBr): 3368.40, 3080.60, 2610.02, 1744.14, 1715.58, 1696.48, 1676.86, 1660.89, 1573.68, 1523.34, 1426.39, 1306.97, 1164.73, 1077.81, 1051.69, 1007.70, 987.58, 943.97, 782.64, 758.60, 707.43, 615.82, 459.15, 441.96 cm^−1^. Elemental analysis calcd. (%) for C_4_H_7_Cl_2_N_7_O (240.07): C 20.01, H 2.94, N 40.85%; found: C 19.97, H 3.01, N 40.92%.

DATOP: DATO (167 mg, 1 mmol) was suspended in 10% HClO_4_ solution (10 mL) and stirred at room temperature for 10 min. Then the colorless solution was volatilized at ambient temperature and pressure, and colorless needle-shaped crystals were observed after one day. The yield is 93%. ^1^H NMR (600 MHz, DMSO-*d*_6_): δ = 8.51, 7.47 ppm. ^13^C NMR (151 MHz, DMSO-*d*_6_): δ = 162.90, 154.02, 148.82, 140.13 ppm. IR (KBr): 3428.09, 3334.85, 1733.89, 1702.42, 1666.45, 1545.08, 1465.06, 1331.88, 1087.35, 987.75, 946.35, 743.86, 688.14, 626.58, 574.25, 490.46, 439.32 cm^−1^. Elemental analysis calcd. (%) for C_4_H_7_Cl_2_N_7_O_9_ (368.07): C 13.05, H 1.92, N 26.64%; found: C 13.01, H 1.97, N 26.56%.

## 3. Results and Discussion

### 3.1. Synthesis

**Scheme 1:**
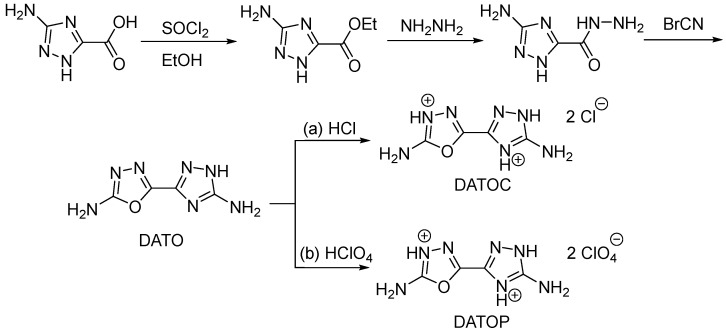
Syntheses of DATOC and DATOP.

DATO was firstly synthesized by Lu’s group [22], and then Dharavath’s group [23] synthesized DATO using an alternate synthetic route. As shown in Scheme 1, DATO was further converted into DATOC by adding hydrochloric acid (HCl, 36%). To construct hydrogen-bonded organic frameworks, perchloric acid (HClO_4_, 10%) was selected to replace hydrochloric acid, which resulted in the self-assembly of triazole–oxadiazole units and oxygen-rich perchlorate anion via hydrogen bonds to yield DATOP. The colorless crystals were obtained from the solution. The structures of these materials were well-characterized by IR spectroscopy, NMR spectroscopy, and elemental analysis, as well as single-crystal X-ray diffraction.

### 3.2. Crystal Structure Analysis

The crystal structures of DATOC and DATOP were determined by X-ray single-crystal diffraction. The detailed crystallographic data for the two crystal structures are listed in the Supplementary Materials.

**Figure 1:**
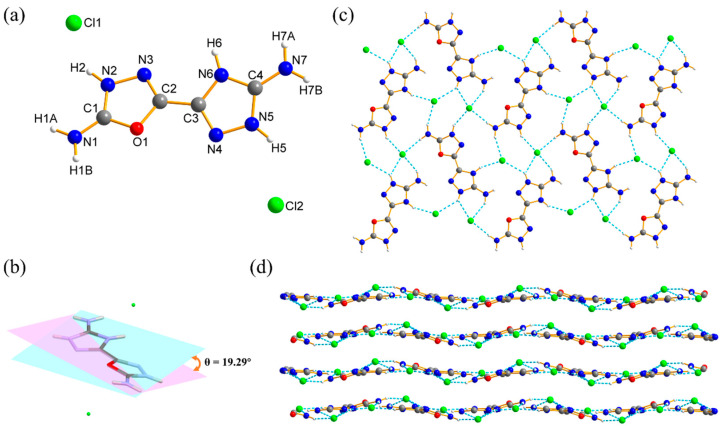
(**a**) Crystal structure of DATOC; (**b**) the planarity of triazole–oxadiazole; (**c**) the layer structure of DATOC; (**d**) “No-linked” layers in DATOC.

Compound DATOC crystallizes in the monoclinic space group *P*2_1_/c with four molecules per unit cell (Z = 4). The crystal structure of DATOC is shown in Figure 1a. The length of the C2-C3 bond connecting the triazole and oxadiazole rings is 1.4381 Å, which is much shorter than the length of the C-C single bond (1.54 Å). Two amino groups are coplanar with their bonded ring, respectively (H7B-N7-C4-N5, 0.229°; H1B-N1-C1-O1, 0.203°). Nevertheless, the oxadiazole and triazole rings form a dihedral angle of 19.29° (Figure 1b). Figure 1c displays the two-dimensional layer structures of DATOC. There are two types of chlorine anions with different bonding modes in DATOC. Cl1 forms three hydrogen bonds with the hydrogen atoms of the amino group and N-H moieties, while Cl2 forms four hydrogen bonds. At the same time, no hydrogen bonds can be observed between layers due to the small size and nature of the chlorine anion (Figure 1d).

**Figure 2:**
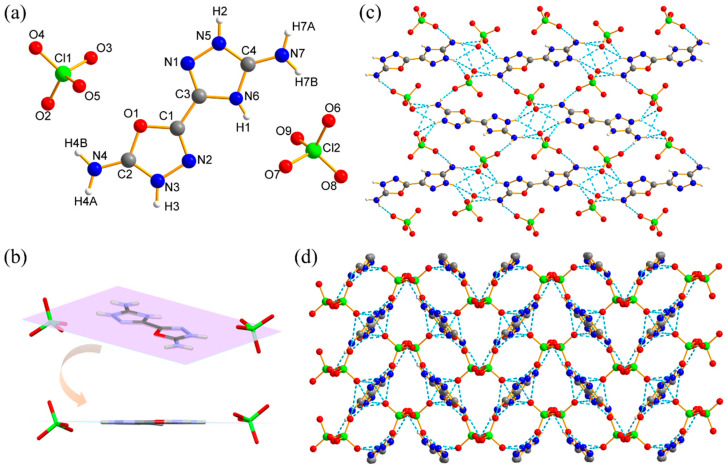
(**a**) Molecular structure of DATOP; (**b**) the coplanar structure of the cation in DATOP; (**c**) the layer structure of DATOP; (**d**) the hydrogen-bonded organic framework in DATOP.

Compound DATOP crystallizes in the monoclinic space group *P*_1_2_1_/c_1_ with two molecules per unit cell (Z = 2). Figure 2a shows the structure of DATOP. The connecting C (triazole)−C (oxadiazole) bond with a length of 1.4355 Å is slightly shorter than that of DATOC. It is worth noting that in addition to the planarity of two amino groups with its bonded ring (H7A-N7-C4-N5, 0.012°; H4B-N4-C2-O1, −0.499°), the torsion angle of 180.000° observed in O1-C1-C3-N6 indicates that triazole and oxadiazole rings are coplanar (Figure 2b). Similarly, the triazole–oxadiazole ion exhibited flatness in its fluoroborate [24]. Additionally, the crystals of DATOP show a wave-like stacking style, which is similar to the fluoroborate of DATO [24]. The triazole–oxadiazole ions exhibit similar planarity, as evidenced by the N3-C2-C3-N4 (178.8°, in DATOC) and the N1-C3-C1-N2 (179.9°, in DATOP). Due to the oxygen-rich perchlorate anion, an intense hydrogen bonding framework can be observed in DATOP (Figure 2c). Each anion is linked with three adjacent cations by seven hydrogen bonds, and thereby, a caged hydrogen bonding framework is formed in two cations and two anions. Different from DATOC, the perchlorate anion is a favorable hydrogen-bonding acceptor, which favors the construction of a hydrogen-bonded organic framework in DATOP (Figure 2d).

### 3.3. Density

**Figure 3:**
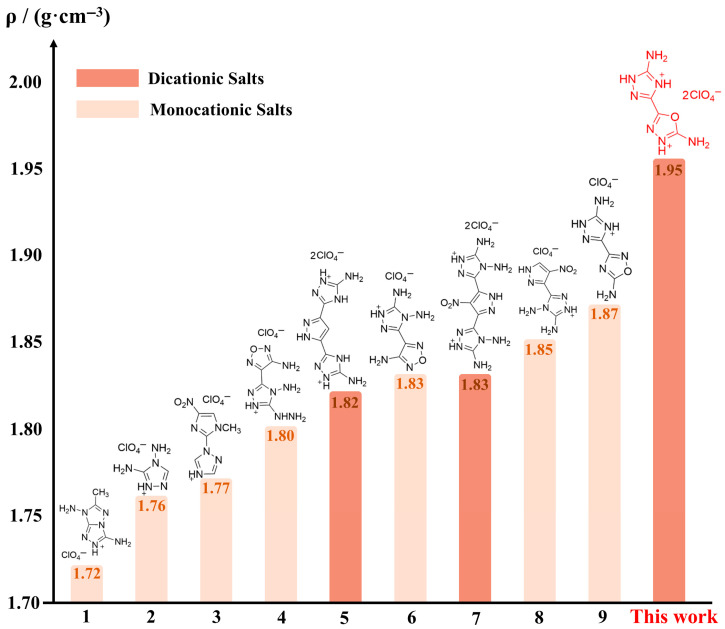
The density of triazole- and oxadiazole-based perchlorates [25,26,27,28,29,30,31,32,33].

Density is a key parameter for energetic materials as the energetic performances of energetic materials are remarkably depend on their density. Significantly, the density of DATOP (*ρ* = 1.954 g·cm^−3^, 298 K) is higher than that of DATOC (*ρ* = 1.717 g·cm^−3^, 298 K) as well as many reported triazole- and oxadiazole-based perchlorates (Figure 3). The intense hydrogen bonding framework of DATOP gives rise to its high density.

**Figure 4:**
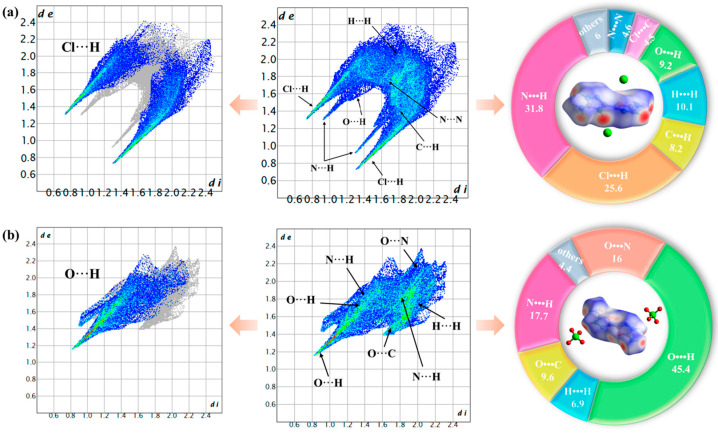
Two-dimensional fingerprint plots and Hirshfeld surface of DATOC (**a**) and DATOP (**b**).

To gain more information on the hydrogen bonding framework and study its influence on density, the Hirshfeld surfaces and 2D fingerprint plots, as well as the percentage contributions of each atom (Figure 4), were calculated and analyzed by employing CrystalExplorer 17 software [34]. In Hirshfeld surfaces, the red and blue areas represent high and low close contact populations, respectively. The thick spike of DATO^2−^ in DATOP reveals that hydrogen bonds are observed, which is in accordance with our previous discussions. Furthermore, the shorter *di* + *de* of the spike suggests more intensive hydrogen bonding interactions in DATOP. The pie graphs show the individual atomic contact percentage contributions to the Hirshfeld surfaces. As shown in the graphs, the O-H (45.4%) interactions in DATOP are stronger than the summation of Cl-H (25.6%) and O-H (9.2%) interactions in DATOC. In a word, the analysis results of Hirshfeld surface analyses demonstrate that DATOP possesses stronger hydrogen bonding interactions than DATOC.

### 3.4. Stabilities

For preliminary safety testing, we measured the mechanism sensitivities of compounds DATOC and DATOP using the standard BAM method. Both DATOC (IS = 28 J) and DATOP (IS = 22 J) are more insensitive to impact stimulus, compared with RDX (IS = 7.4 J) and TNT (IS = 15 J). For friction sensitivity, DATOC (FS = 280 N) and DATOP (FS = 210 N) are superior to RDX (FS = 120 N). To study the thermal stabilities of these materials, differential scanning calorimetric (DSC) was performed at 10 K·min^−1^ under a nitrogen flow in the temperature range of 50–400 °C. From the DSC curves (Figure S5), DATOP (*T_d_* = 273 °C) exhibits a noteworthy enhancement in initial decomposition temperature in comparison with DATOC (*T_d_* = 154 °C). Moreover, the thermal stability of DATOP is superior to RDX (*T_d_* = 204 °C) and comparable to HMX (*T_d_* = 275 °C).

**Figure 5:**
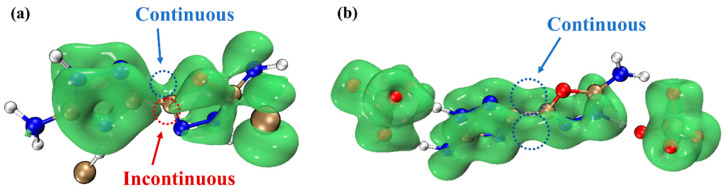
The π–electron density distribution diagram of DATOC (**a**) and DATOP (**b**).

The aromaticity is an internal factor of thermal stability. Localized orbital locator π of DATOC and DATOP was carried out to better study their aromaticity. The LOL-π isosurface maps [35], as illustrated in Figure 5, provide a visual representation of the distribution of π electrons within the molecules of DATOC and DATOP. In comparison, DATOP possesses larger areas of π–electron distribution compared to DATOC. More importantly, the planar triazole–oxadiazole skeleton leads to a closed π-electron ring in DATOP, while a breakage of π-electron can be observed around the C-C bond connecting the triazole and oxadiazole rings in DATOC, which indicates that DATOP is aromatic and supports the result of the higher thermal stability of DATOP.

### 3.5. Detonation Performances

The heats of formation (HOF) were calculated by using the Gaussian 09 (Revision A.02) [36] suite of programs. The HOFs of DATOC and DATOP were −80.3 and −62.6 kJ·mol^−1^, respectively, both of which are higher than that of TNT (HOF = −115.0 kJ·mol^−1^). Benefiting from its oxygen-rich anion, the oxygen balance of DATOP (OB = −6.5%) is dramatically higher than that of DATOC (OB = −63.3%) and close to zero oxygen balance. Their detonation performances were evaluated by using EXPLO 5 [37]. Their physicochemical properties have been listed in Table 1. DATOC (*D* = 5984 m·s^−1^, *P* = 12.4 GPa) shows a relatively low energetic performance. DATOP exhibits remarkable increases of 2640 m·s^−1^ and 22 GPa in detonation velocity and pressure, respectively, which certifies the advantage of perchlorate anion in improving the density and oxygen balance and thereby boosting its energetic performance. Meanwhile, the detonation velocity of DATOP (*D* = 8624 m·s^−1^) is superior to TNT (*D* = 6881 m·s^−1^) and close to RDX (*D* = 8795 m·s^−1^).

## 4. Conclusions

In summary, a salt formation strategy was employed to balance energy and stability in energetic materials. The introduction of oxygen-rich perchlorate anion resulted in higher density and oxygen balance. Compared to DATOC (*ρ* = 1.717 g·cm^−3^, *T_d_* = 154 °C), both the density and thermal stability of DATOP (*ρ* = 1.954 g·cm^−3^, *T_d_* = 273 °C) were simultaneously improved. Due to its higher density, DATOP (*D* = 8624 m·s^−1^, *P* = 34.4 GPa) exhibits significant enhancements in detonation performances compared to DATOC (*D* = 5984 m·s^−1^, *P* = 12.4 GPa). Meanwhile, compared with DATOC (IS = 28 J, FS = 280 N), DATOP (IS = 22 J, FS = 210 N) shows comparable sensitivities toward impact and friction. These desired results revealed that the strategy of salt formation provides a new path for the modulation of energy and safety in energetic materials.

## Figures and Tables

**Table 1:** Physicochemical properties of DATOC and DATOP.

Compounds	*T_d_* ^a^ (°C)	*ρ* ^b^ (g·cm^−3^)	∆H_f_ ^c^ (kJ·mol^−1^)	D ^d^ (m·s^−1^)	P ^e^ (GPa)	IS ^f^ (J)	FS ^g^ (J)	OB ^h^ (%)
DATOC	154	1.717	−80.3	5984	12.4	28	280	−63.3
DATOP	273	1.954	−62.6	8624	34.4	22	210	−6.5
TNT ^i^	295	1.65	−355.0	6881	19.5	15	353	−74.0
RDX ^i^	204	1.81	80.0	8795	34.9	7.4	120	−21.6

^a^ Thermal decomposition temperature (DSC, onset, 10 °C·min^−1^). ^b^ Recalculated from low-temperature X-ray densities (*ρ*_298K_ = *ρ*_T_/(1 + α_V_(298-T_0_); α_V_ = 1.5 × 10^−4^ K^−1^). ^c^ Heat of formation. ^d^ Detonation velocity. ^e^ Detonation pressure. ^f^ Impact sensitivity. ^g^ Friction sensitivity. ^h^ Oxygen balance. ^i^ Ref. [38].

## Data Availability

The original contributions presented in this study are included in the article/Supplementary Materials. Further inquiries can be directed to the corresponding authors.

## Supplementary Materials

The following supporting information can be downloaded at: https://www.mdpi.com/article/10.3390/ma18153435/s1. Table S1: Crystallographic data for DATOC and DATOP; Tables S2–S5: Bond lengths [Å], angles [°], and hydrogen bonds of DATOC and DATOP; Figures S1 and S2: Molecular structure of DATOC and DATOP; Figure S3: DSC Plot of DATOC and DATOP; Figure S4: TG-DSC Plot of DATOP; Figures S5–S10: NMR and IR spectra of DATOC and DATOP; Figure S11: Isodesmic equation of DATOC and DATOP. References [36,39,40] are cited in the Supplementary Materials.
