# Supplementary material for: Salt Formation of the Alliance of Triazole and Oxadiazole Towards Balanced Energy and Safety
Source: Materials (Basel). 2025 Jul 22;18(15):3435. doi: 10.3390/ma18153435 (PMC12347762; doi:10.3390/ma18153435)
Supplement: Supplementary file 1 [file materials-18-03435-s001.zip › materials-3645828-supplementary.pdf]

Supplementary materials

Construction of hydrogen-bonded organic frameworks: promising strategy for regulation of energy and stability in energetic materials

Yang Liu [1,+], Meiqi Wang [1,+], Jiawei Men [1], Bibo Li [1], Shangbiao Feng [1], Shuangfei Zhu [1], Guangrui Liu [1], Ruijun Gou [1,*], Shuhai Zhang [1,*], Ming Lu [2] and Li Yang [3]

[1] School of Environmental and Safety Engineering, North University of China, Taiyuan 030051, China

[2] School of Chemistry and Chemical Engineering, Nanjing University of Science and Technology, Nanjing 210094, China

[3] Shanxi Jiangyang Chemical Limited Company, Taiyuan 030041, Shanxi, China

[+] These authors contributed equally to this work

[*] Correspondence: grjzsh@163.com (R.G.); zsh93y@nuc.edu.cn (S.Z.)

Table of contents

1. General methods
2. X-ray crystallography detail
3. DSC and TG-DSC curves
4. Elementary analysis, NMR and IR spectroscopy
5. Theoretical study
6. References

1. General methods

All reagents for this experiment were purchased from commercial sources and used without any further purification. NMR spectra (^{1}H and ^{13}C) were recorded on a ADance III 600MH nuclear magnetic resonance spectrometer, and by using dimethyl sulfoxide (DMSO-d_6) as a locking solvent. FT-IR spectra were obtained on a DTGS KBr spectrometer. The thermal decomposition behavior of these compounds were tested using differential scanning calorimetry (DSC) and at heating rate of 10 °C·min^{-1}. DSC test was determined using a DSC-800B apparatus under a nitrogen atmosphere at a flow rate of 50 mL·min^{-1} from ambient temperature to 400 °C. Impact and friction sensitivities for **DATOC** and **DATOP** were performed on a BAM fall hammer BFH-12 and a BAM friction apparatus FSKM-10, respectively.

2. X-ray crystallography detail

In order to determine the single crystal structure of **DATOC** and **DATOP**, crystals of **DATOC** and **DATOP** suitable for single crystal X-ray diffraction were obtained by slow evaporation from deionized water solution. At the diffraction data were collected using a graphite monochromator of Cu-Kα alpha rays (λ = 1.54184 Å) on the HyPix diffractometer using a ROD and Synergy custom systems. Data collection and initial unit cell refinement were performed with CrysAlisPro 1.171.42.93a. Data reduction were performed by using SHELXL (2018/3). The structures were solved by direct methods and refined by the full matrix least-squares based on F2 using SHELXT and SHELXS (v2008/1) program package. The full-matrix least-squares refinement on F^2 involved atomic coordinates and anisotropic thermal parameters for all non-H atoms. All non-hydrogen atoms were refined anisotropically. The hydrogen atoms attached to ligands were generated geometrically and refined using a riding model.

Table S1. Crystallographic data of **DATOC** and **DATOP**.

Compound	DATOC	DATOP
CCDC	2382202	2382194
Empirical formula	$C_4H_7Cl_2N_7O$	$C_4H_7Cl_2N_7O_9$
Formula weight [g·mol^{-1}]	240.07	368.07
Temperature [K]	149.99(10)	286.0(5)
Wavelength [Å]	1.54184	1.54184

Crystal system	Monoclinic	Monoclinic
Space group	$P2_1/c$	$P2_1/c$
Z	4	2
Crystal size [mm^3]	0.06 × 0.04 × 0.03	0.07 × 0.05 × 0.04
a [Å]	10.8659(6)	5.1720(10)
b [Å]	12.7506(6)	11.0401(2)
c [Å]	6.6936(3)	11.0029(2)
α [°]	90	90
β [°]	101.555(5)	96.370(2)
γ [°]	90	90
Volume [$Å^3$]	908.57(8)	624.38(2)
ρ [g/cm^3]	1.755	1.958
μ [mm^{-1}]	6.322	5.379
F (000)	488.0	372.0
θ range [°]	4.1530-77.2940	4.0180-76.3780
Index ranges	$-13 \leq h \leq 13$, $-16 \leq k \leq 16$, $-7 \leq l \leq 8$	$-4 \leq h \leq 6$, $-11 \leq k \leq 13$, $-13 \leq l \leq 13$
Goodness-of-fit on F^2	1.056	1.120
Data / restraints / parameters	3267/0/128	1222/0/105
R_1 [I >2sigma (I)]	0.0486	0.0446
wR_2 [I >2sigma (I)]	0.1373	0.1157
R_1 [all data]	0.0528	0.0461
wR_2 [all data]	0.1415	0.1166

Table S2. Bond lengths [Å] and angles [°] for **DATOC**.

O1-C1	1.347(3)	C1-O1-C2	103.7(2)
O1-C2	1.376(3)	H1A-N1-H1B	120.0

N1-H1A	0.8800	C1-N1-H1A	120.0
N1-H1B	0.8800	C1-N1-H1B	120.0
N1-C1	1.295(4)	N3-N2-H2	124.9
N2-H2	0.8800	C1-N2-H2	124.9
N2-N3	1.387(3)	C1-N2-N3	110.2(2)
N2-C1	1.330(4)	C2-N3-N2	103.4(2)
N3-C2	1.276(4)	C3-N4-N5	103.8(2)
N4-N5	1.376(3)	N4-N5-H5	124.5
N4-C3	1.307(4)	C4-N5-N4	111.1(2)
N5-H5	0.8800	H7A-N7-H7B	120.0
N5-C4	1.348(4)	C4-N7-H7A	120.0
N6-H6	0.8800	C4-N7-H7B	120.0
N6-C3	1.369(4)	N1-C1-O1	122.3(3)
N6-C4	1.339(4)	N1-C1-N2	129.5(3)
N7-H7A	0.8800	N2-C1-O1	108.2(2)
N7-H7B	0.8800	O1-C2-C3	119.6(2)
N7-C4	1.324(4)	N3-C2-O1	114.4(2)
C2-C3	1.438(4)	N3-C2-C3	126.0(3)
		N4-C3-N6	112.1(2)
		N4-C3-C2	126.0(3)

Table S3. Hydrogen bonds in **DATOC**.

D-H···A	D-H/Å	H···A/Å	D···H/ Å	D-H···A/°
N1-H1A···Cl2	0.88	2.31	3.154	161
N2-H2···Cl1	0.88	2.21	2.989	147
N1-H1B···Cl1	0.88	2.26	3.096	159
N7-H7A···N4	0.88	2.35	3.125	147
N7-H7B···Cl1	0.88	2.38	3.140	145
N5-H5···Cl1	0.88	2.55	3.232	135

N5-H5···Cl2	0.88	2.50	3.181	135
N6-H6···Cl2	0.88	2.18	3.005	157

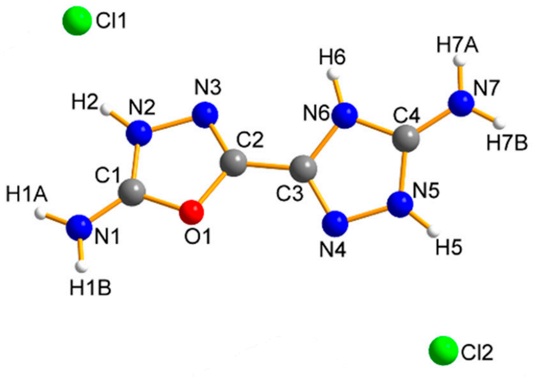

Figure S1. Molecular structure of **DATOC**.

Table S4. Bond lengths [Å] and angles [°] for **DATOP**.

Cl1-O2	1.421(3)	O2-Cl1-O3	109.9(2)
Cl1-O3	1.422(3)	O2-Cl1-O5	109.47(17)
Cl1-O4	1.421(2)	O3-Cl1-O5	106.91(18)
Cl1-O5	1.429(2)	O4-Cl1-O2	109.76(17)
O1-C2	1.342(4)	O4-Cl1-O3	110.3(2)
O1-C1	1.373(3)	O4-Cl1-O5	110.50(16)
O1-H1	0.78(6)	C2-O1-C1	105.5(2)
C1-C1	1.436(5)	C2-O1-H1	130(4)
N1-C2	1.342(4)	C1-O1-H1	124(4)
N1-C1	1.373(3)	C2-N1-C1	105.5(2)
N1-H1	0.78(6)	C2-N1-H1	130(4)
N2-N3	1.374(4)	C1-N1-H1	124(4)
N2-C1	1.286(4)	C1-N2-N3	103.1(2)
N3-H3	0.8600	N2-N3-H3	124.0
N3-C2	1.325(4)	C2-N3-N2	111.9(2)
N4-H4A	0.8600	C2-N3-H3	124.0
N4-H4B	0.8600	H4A-N4-H4B	120.0

N4-C2	1.299(4)	C2-N4-H4A	120.0
		C2-N4-H4B	120.0
		N3-C2-O1	106.4(3)
		N3-C2-N1	106.4(3)
		N4-C2-O1	124.1(3)
		N4-C2-N1	124.1(3)
		N4-C2-N3	129.5(3)
		O1-C1-C1	120.5(3)
		N1-C1-C1	120.5(3)
		N2-C1-O1	113.0(2)
		N2-C1-N1	113.0(2)

Table S5. Hydrogen bonds in **DATOP**.

D-H···A	D-H/Å	H···A/Å	D···H/ Å	D-H···A/°
O1-H1···O3	0.78	2.35	3.120	171
N3-H3···O5	0.86	2.17	2.891	141
N3-H3···O4	0.86	2.40	3.048	132
N4-H4A···O4	0.86	2.39	3.068	136
N4-H4A···O5	0.86	2.35	3.075	142
N4-H4B···O2	0.86	2.14	2.984	166

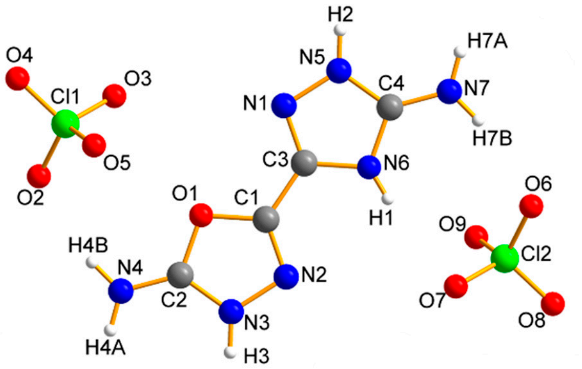

Figure S2. Molecular structure of **DATOP**.

3. DSC and TG-DSC curves

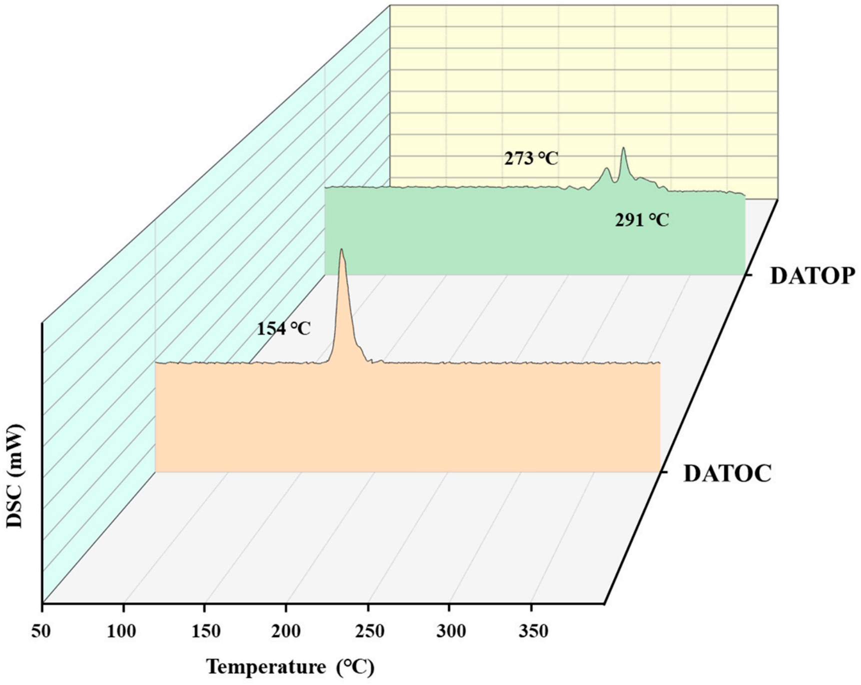

Figure S3. DSC Plot of **DATOC** and **DATOP**.

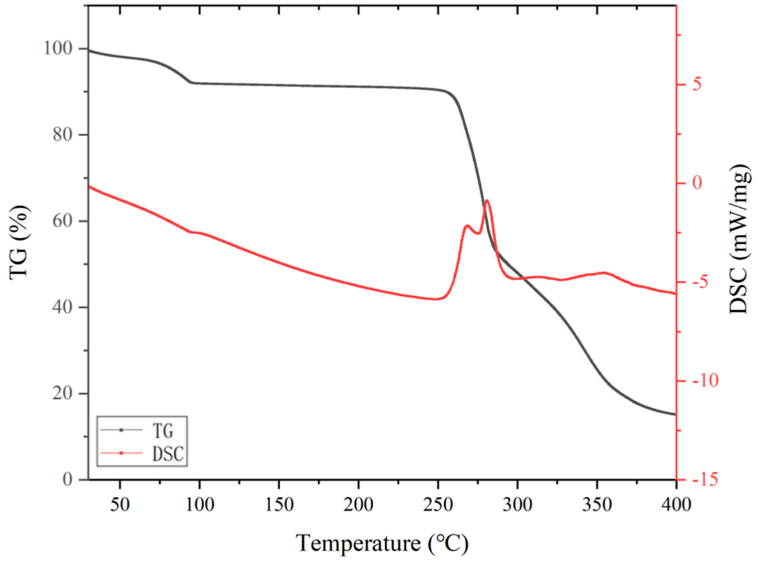

Figure S4. TG-DSC Plot of **DATOP**.

4. Elementary analysis, NMR and IR spectroscopy

DATOC: ^{1}H NMR (600 MHz, DMSO-d_6): δ = 8.35 ppm. ^{13}C NMR (151 MHz, DMSO-d_6): δ = 162.63, 154.51, 149.05, 140.57 ppm. IR (KBr): 3368.40, 3080.60, 2610.02, 1744.14, 1715.58, 1696.48, 1676.86, 1660.89, 1573.68, 1523.34, 1426.39, 1306.97, 1164.73, 1077.81, 1051.69, 1007.70, 987.58, 943.97, 782.64, 758.60, 707.43, 615.82, 459.15, 441.96 cm^{-1}. Elemental analysis

calcd. (%) for $C_4H_7Cl_2N_7O$ (240.07): C 20.01, H 2.94, N 40.85 %; found: C 19.97, H 3.01, N 40.92 %.

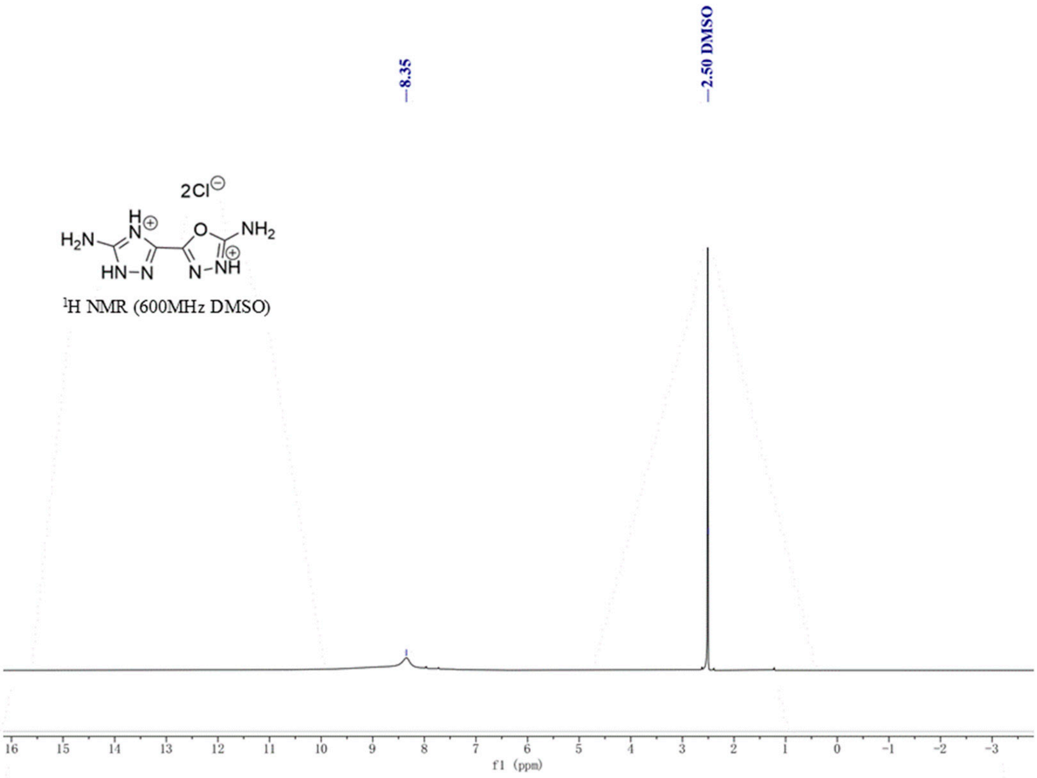

Figure S5. ^{1}H NMR spectra in DMSO-d_6 for **DATOC**.

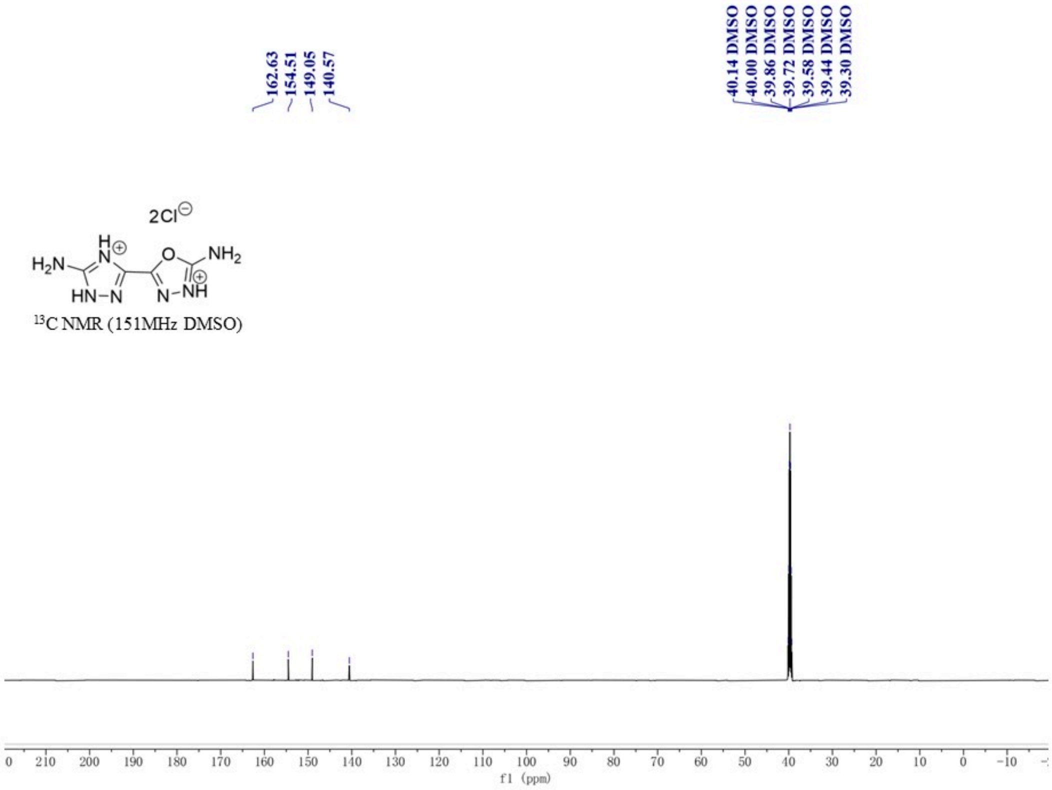

Figure S6. ^{13}C NMR spectra in DMSO-d_6 for **DATOC**.

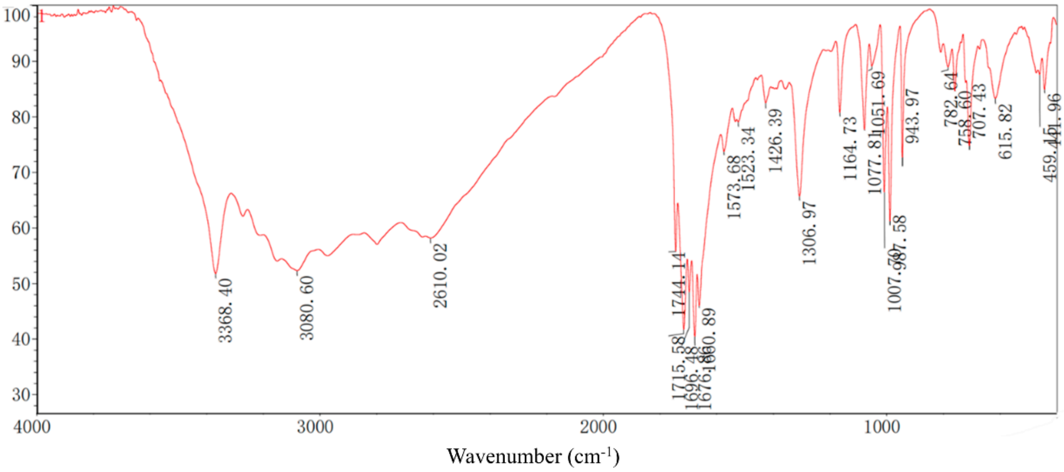

Figure S7. IR Spectra of **DATOC**.

DATOP: ^{1}H NMR (600 MHz, DMSO-d_6): δ = 8.52, 7.48 ppm. ^{13}C NMR (151 MHz, DMSO-d_6): δ = 162.90, 154.02, 148.82, 140.13 ppm. IR (KBr): 3428.09, 3334.85, 1733.89, 1702.42, 1666.45, 1545.08, 1465.06, 1331.88, 1087.35, 987.75, 946.35, 743.86, 688.14, 626.58, 574.25, 490.46, 439.32 cm^{-1}. Elemental analysis calcd. (%) for $C_4H_7Cl_2N_7O_9$ (368.07): C 13.05, H 1.92, N 26.64 %; found: C 13.01, H 1.97, N 26.56 %.

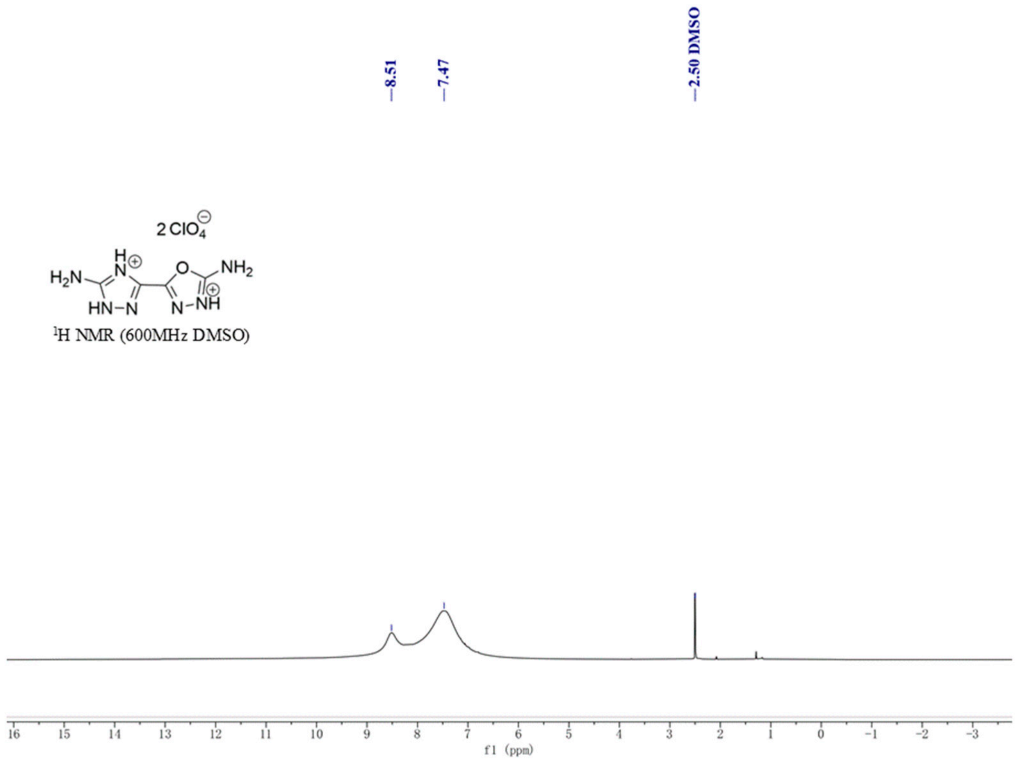

Figure S8. ^{1}H NMR spectra in DMSO-d_6 for **DATOP**.

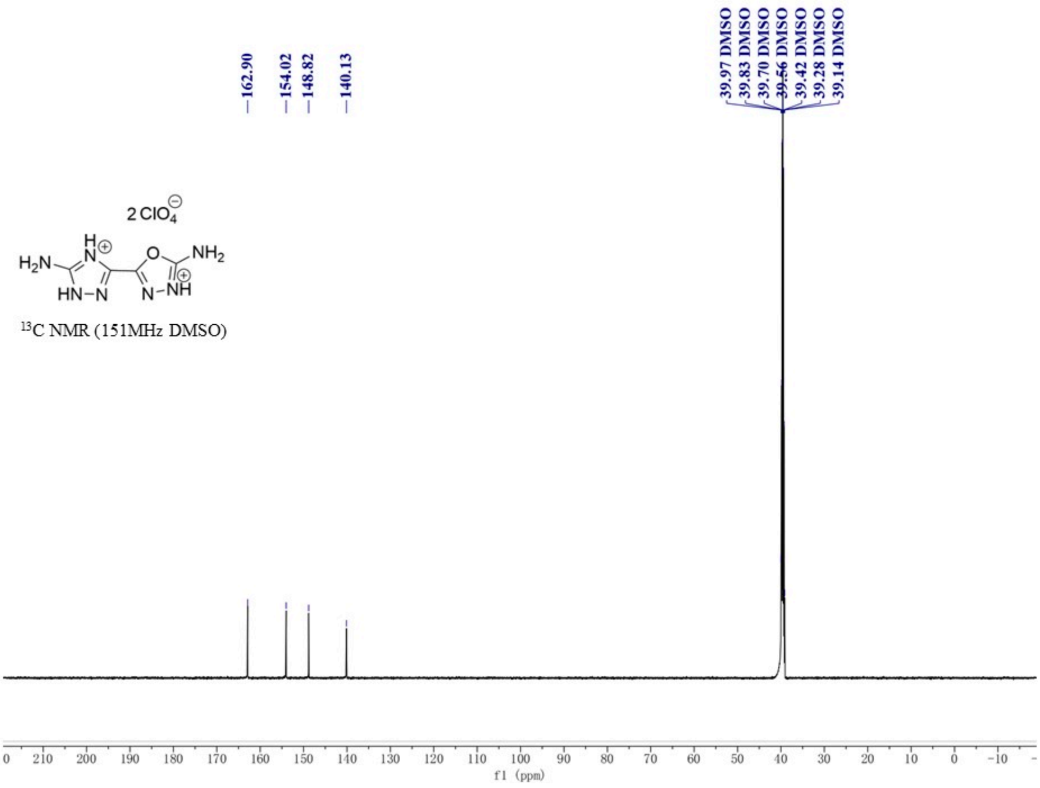

Figure S9. ^{13}C NMR spectra in DMSO-d_6 for **DATOP**.

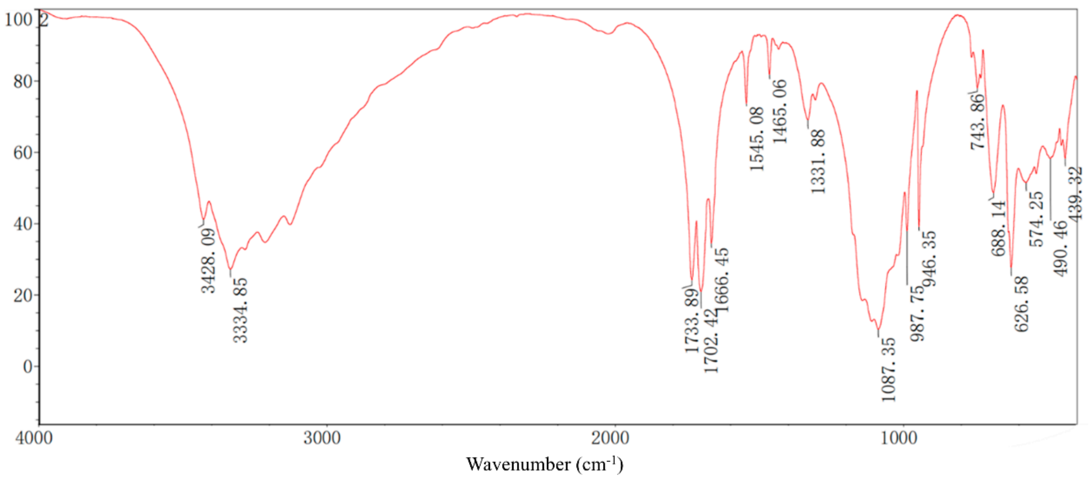

Figure S10. IR Spectra of **DATOP**.

5. Theoretical study

All of the ab initio calculations involved in this work were carried out using the Gaussian 09 suite of programs[1]. The geometric optimization and frequency analysis of the structures are based on available single-crystal structures and using the B3LYP functional with the 6-311++G (d, p) basis set. The geometrical were optimized with no constraints imposed under default convergence criteria. The formation enthalpies of the corresponding cations of **DATOC** and **DATOP** were calculated using equibond equation. Total energy (E0) and zero-point energy (ZPE) were calculated with vibrational frequency analysis. The heats of formation were obtained by using the isodesmic reaction approach. Atomization energies were obtained by employing the G2 ab initio method. All the optimized structures were characterized to be true local energy minima on the potential energy surface without imaginary frequencies.

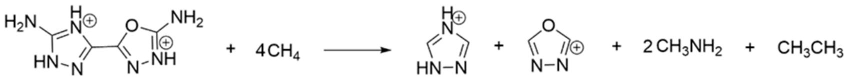

Figure S11. Isodesmic equation of **DATOC** and **DATOP**

The solid-phase heat of formation is calculated based on a Born-Haber energy cycle[2]. The number is simplified by equation 1:

$$\Delta H_f^0\ (298K) = \Delta H_f^0\ (cation, 298K) + \Delta H_f^0\ (anion, 298K) - \Delta H_L \qquad (1)$$

where ΔH_L is the lattice energy, which could be predicted by using the formula suggested by Jenkins et al[2]. [Eq. (2)]

$$\Delta H_L = U_{POT} + [p(n_M/2 - 2)] + q(n_X/2 - 2)]RT \qquad (2)$$

where n_M and n_X depend on the nature of the ions, M^{q+} and X^{p-}, respectively. The equation for lattice potential energy U_{POT} [Eq. (3)] has the form:

$$U_{POT}\ [kJ\ mol^{-1}] = \gamma(\rho_m/M_m)^{1/3} + \delta \quad (3)$$

where ρ_m [g cm^{-3}] is the density, M_m is the chemical formula mass of the ionic material, and values for (g) and the coefficients γ (kJ mol^{-1} cm) and δ (kJ mol^{-1}) are assigned literature values.

The solid-state enthalpy of formation for neutral compound can be estimated by subtracting the heat of sublimation from gas-phase heat of formation. Based on the literature[3], the heat of sublimation can be estimated with Trouton's rule according to supplementary equation 1, where T represents either the melting point or the decomposition temperature when no melting occurs prior to decomposition:

$$\Delta H_{sub} = 188/J\ mol^{-1}K^{-1} \times T$$

For $C_aH_bN_cO_dCl_e$ energetic materials, the calculation equation of oxygen balance is $OB(\%) = \frac{(d-2a-\frac{b}{2}+e)}{M_w} \times 1600$, where M_w is formula weight, g·mol^{-1}.

6. References

1. Frisch, M.J.; Trucks, G.W.; Schlegel, H.B.; Scuseria, G.E.; Robb, M.A.; Cheeseman, J.R.; Zakrzewski, V.G.; Montgomery, J.A.; Stratmann, R.E.; Burant, J.C.; Dapprich, S.; Millam, J.M.; Daniels, A.D.; Kudin, K.N.; Strain, M.C.; Farkas, O.; Tomasi, J.; Barone, V.; Cossi, M.; Cammi, R.; Mennucci, B.; Pomelli, C.; Adamo, C.; Clifford, S.; Ochterski, J.; Petersson, G.A.; Ayala, P.Y.; Cui, Q.; Morokuma, K.; Malick, D.K.; Rabuck, A.D.; Raghavachari, K.; Foresman, J.B.; Cioslowski, J.; Ortiz, J.V.; Baboul, A.G.; Stefanov, B.B.; Liu, G.; Liashenko, A.; Piskorz, P.; Komaromi, I.; Gomperts, R.; Martin, R.L.; Fox, D.J.; Keith, T.; Laham, M.A.; Peng, C.Y.; Nanayakkara, A.; Gonzalez, C.; Challacombe, M.; Gill, P.M.W.; Johnson, B.; Chen, W.; Wong, M.W.; Andres, J.L.; Gonzalez, C.; Head-Gordon, M.; Replogle, E.S.; Pople, J.A. *Gaussian 09*, revision A.02, Gaussian, Inc., Wallingford, CT, **2009**.
2. Jenkins, H.D.B.; Tudela, D.; Glasser, L. Lattice potential energy estimation for complex ionic salts from density measurements. *Inorg. Chem.* **2002**, *41*, 2364-2367.
3. Westwell, M.S.; Searle, M.S.; Wales, D.J. Empirical correlations between thermodynamic properties and intermolecular forces. *J. Am. Chem. Soc.* **1995**, *117*, 5013-5015.
